# Cellular and molecular basis of cerebellar development

**DOI:** 10.3389/fnana.2013.00018

**Published:** 2013-06-26

**Authors:** Salvador Martinez, Abraham Andreu, Nora Mecklenburg, Diego Echevarria

**Affiliations:** ^1^Experimental Embryology Lab, Consejo Superior de Investigaciones Científicas, Instituto de Neurociencias de Alicante, Universidad Miguel HernandezAlicante, Spain; ^2^Department of Neuroscience, Max-Delbrück-Center for Molecular MedicineBerlin, Germany

**Keywords:** rostral hindbrain, caudal mesencephalon, cerebellum, isthmus, isthmic organizer, isthmic constriction, Fgf8, morphogenesis

## Abstract

Historically, the molecular and cellular mechanisms of cerebellar development were investigated through structural descriptions and studying spontaneous mutations in animal models and humans. Advances in experimental embryology, genetic engineering, and neuroimaging techniques render today the possibility to approach the analysis of molecular mechanisms underlying histogenesis and morphogenesis of the cerebellum by experimental designs. Several genes and molecules were identified to be involved in the cerebellar plate regionalization, specification, and differentiation of cerebellar neurons, as well as the establishment of cellular migratory routes and the subsequent neuronal connectivity. Indeed, pattern formation of the cerebellum requires the adequate orchestration of both key morphogenetic signals, arising from distinct brain regions, and local expression of specific transcription factors. Thus, the present review wants to revisit and discuss these morphogenetic and molecular mechanisms taking place during cerebellar development in order to understand causal processes regulating cerebellar cytoarchitecture, its highly topographically ordered circuitry and its role in brain function.

## Introduction

The vertebrate brain is a remarkably complex anatomical structure that contains diverse subdivisions and neuronal subtypes with specific, sometimes prodigal, synaptic connections that contribute to the complexity of its function. During development the primordial brain (the neural tube) has to be progressively regionalized. A precise spatial and temporal arrangement of gene expression regulates intercellular and intracellular signals driving a proper molecular patterning that is required for this regionalization. Pioneering genoarchitectural studies and fate mapping experiments established correlations on how morphogens, transcription factors, and other signaling molecules modulate the specification of neuroepithelial territories, to generate the structural complexity and cellular diversity that characterizes the brain (revised in Puelles and Rubenstein, 2003; Martínez et al., 2012; Puelles and Ferran, 2012). Thus, the combination of molecular genetics (gene expression maps) and modern neuroanatomy (based on histochemistry and highly sensitive neuroimaging) have led to an increased interest in describing the neurodevelopmental mechanisms underlying structural disorders and intellectual discapacities that we currently observe in congenital anomalies of the human brain.

Among the classical systems used to study the structure and function of the central nervous system the cerebellum has steadily gained popularity and has become one of the most experimentally tractable systems in the brain. Much of our knowledge about structure, function, and development of the mouse cerebellum was achieved by studying spontaneous mutations (Sotelo, 2004), but also by using sophisticated genetic tools allowing a more precise and mechanistic level of analysis (Joyner and Sudarov, 2012; Tvrdik and Capecchi, 2012).

This review focuses on the basic developmental biology of the cerebellum starting from morphological features in order to distinguish the origin and specification of the cerebellar neuroepithelial anlage, as well as to describe the molecular mechanisms implicated in the development of its architectural morphology, stereotyped cellular differentiation, and neuronal distribution. Finally, we summarize relevant works in correlations with those findings in developmental cerebellar disorders of the human cerebellum (Barkovich, 2012).

## The topography and topology of the cerebellar anlage

**Figure 1:**
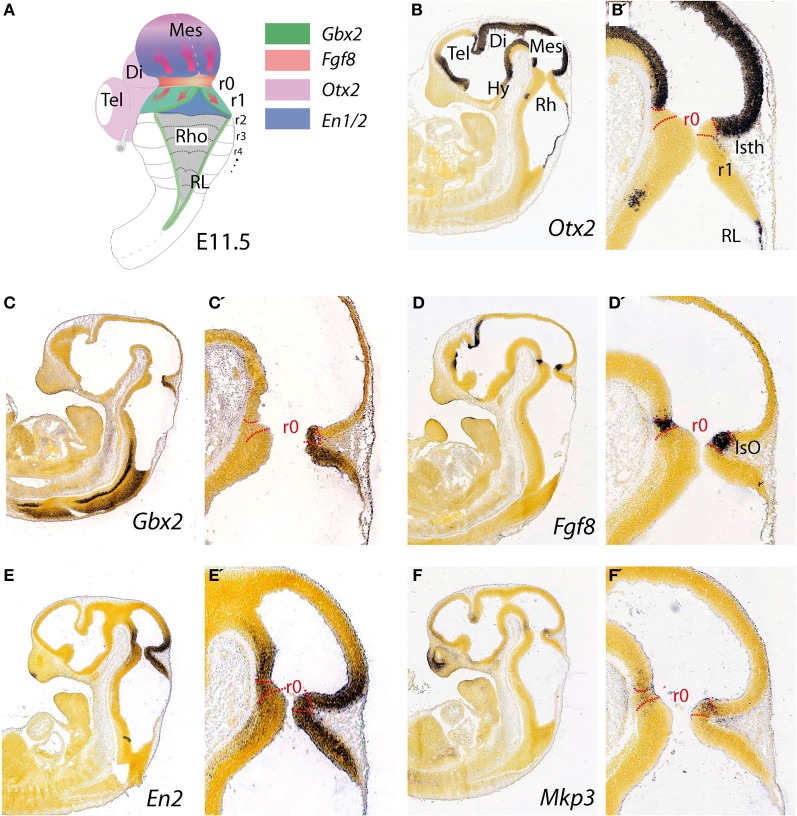
**Topographical location and main molecular characterization of the mid-hindbrain boundary at E11.5. (A)** A dorsal view of an E11.5 mouse embryo illustrating the isthmic constriction (isth) located between the mesencephalon and rhombomere 1 (r1). Moreover, rhombomeres, r0 and r1, which give rise to the cerebellum are highlighted in **(A)**. The different color codes depict the expression pattern of the most important genes related to the morphogenetic activity and the capacity of the IsO (We only consider *Fgf8*-positive territory at this constriction as IsO; see also Martínez, 2001). Expression patterns of genes that are illustrated in **(A)** and their boundaries are also shown by *in situ* hybridization (ISH) at E11.5 for *Otx2* **(B,B′)**, *Gbx2* **(C,C′)**, *Fgf8* **(D,D′)** *En2* **(E,E′)**, and *Mkp3* **(F,F′)**. Panels (**B–F′**) were taken from Allen Institute for Brain Science public resources (http://www.brain-map.org/).

The CNS arises from an apparent homogenous sheet of epithelial cells, the neural plate, induced during gastrulation by the dorsal lip of the blastopore in amphibians (Spemann and Mangold, 1924) or by the Hensen's node in amniotes. During the process of neural induction the neural plate pursues morphological differentiation, its edges thicken and roll up, to close dorsally in order to form the neural tube. The most anterior portion of the neural tube is undergoing drastic changes during early development generating, by differential proliferation, the three primary brain vesicles: the forebrain (prosencephalon), midbrain (mesencephalon), and hindbrain (rhombencephalon); caudal neural tube remains with a cylindrical shape and generates the spinal cord (Martínez and Puelles, 2000). The discovery that putative regulatory genes are expressed in regionally restricted patterns in the developing neural tube has provided new tools for defining histogenic domains and their boundaries at higher resolution. In the rhombencephalon, the segments are termed rhombomeres (r) that from anterior to posterior are known as r0 (the isthmus) and r1–r7, followed by the pseudorhombomeres r8–r11 (Marín and Puelles, 1995; Cambronero and Puelles, 2000; Figure 1A). The mature cerebellum is composed of two cerebellar hemispheres and the vermis located between these hemispheres. Embryonically the cerebellar hemispheres and the vermis originate from the first two rhombomeres. The vermis is part of the alar r0 and roof plates of r0 and r1, whereas the hemispheres belong to the alar r1 (Figures 1A, 3A,B). Already in 1890, Wilhelm His proposed the alar neuroepithelium of the anterior rhombencephalon (or metencephalon) as the origin of the cerebellum. He postulated that from these paired plates, the cerebellum evolves as a bilateral organ, which would subsequently fuse at the dorsal roof midline in a rostral-to-caudal direction, to form a uniform primordium.

The topographical boundary between the mesencephalon and rhombencephalon is the isthmic constriction or simply the isthmus (Isth; Figure 1B′). This was initially interpreted to bridge the midbrain-hindbrain boundary (Alvarado-Mallart, 1993), but is now thought to co-localize with the prospective isthmic territory (r0), as defined early on by the expression of the well-known secreted molecule fibroblast growth factor 8 (*Fgf8*; Crossley et al., 1996). Homotopic and isochronic quail-chick grafting experiments performed in the late 1980's and the 1990's consistently showed that the caudal part of the early midbrain vesicle had a peculiar morphogenesis and generated the rostral and medial part of the prospective cerebellum (Martinez and Alvarado-Mallart, 1989; Hallonet et al., 1990; Alvarez-Otero et al., 1993; Marin and Puelles, 1994; Hidalgo-Sánchez et al., 2005). Therefore, the anterior vermal part of the presumptive cerebellum, instead to result from fusion of lateral cerebellar plates (His, 1889), originated from the caudal and alar portion of the mesencephalic vesicle. Hence, Puelles and collaborators argued that the early mid-hindbrain constriction observed by Vaage (1969) was not a fixed non-proliferative neuroepithelial structure, as proper interneuromeric landmarks are, but it was a wave-like transient conformation of the local proliferating neuroepithelium. In avian embryos gene expression pattern analysis has proved that the relative position of the actual midbrain-hindbrain boundary, is located at the edge of *Otx2* and *Gbx2* expression domains (initially inside mesencephalic vesicle) and moves caudally after stage HH15, to coincide with the pre-existent midbrain hindbrain constriction at around HH20–21 (Martinez and Alvarado-Mallart, 1989; Alvarez-Otero et al., 1993; Hidalgo-Sánchez et al., 2005; Figures 1B,C).

The homeodomain transcription factors of Engrailed family *En1* and *En2* (Figures 1E,E′) are expressed early on in cerebellar and mesencephalic primordial neuroepithelium and both are involved in the formation of the mesencephalic tectum and cerebellum (Figure 1A). Thus, mouse *En1* mutants lack most of the tectum and cerebellum and die at birth, whereas *En2* mutants are viable with a smaller cerebellum and foliation defects (Joyner et al., 1991; Hanks et al., 1995). Experimental studies indicate that the severeness of *En1* and *En2* phenotypes differs due to a relatively early onset of *En1* expression compared to the onset of *En2* expression, rather than differences in protein function (Joyner et al., 1991; Millen et al., 1995). Studies on conditional mutant alleles of *En1* and/or *En2* demonstrated that *En1* is required for cerebellar development only before embryonic day 9, but plays a substantial role in forming the tectum. In fact, *En2* was found to be more potent than *En1* in cerebellar development (Sgaier et al., 2007). In addition these authors proved that there is an *En1/2* dose-dependent genetic subdivision of the tectum into its two functional alar subdivisions (anterior and posterior colliculi) and of the medio-lateral cerebellum into four regions that have distinct molecular coding and represent functional domains.

## The molecular specification of the cerebellar anlage: the isthmic organizer

Distinct neural and glial identities are acquired by neuroepithelial progenitor cells through progressive restriction of histogenetic potential under the influence of local environmental signals. Evidence for morphogenetic regulatory processes at specific locations of the developing neural primordium has led to the concept of secondary organizers, which regulate the identity and regional polarity of neighboring neuroepithelial regions (Ruiz i Altaba, 1998; for review see Echevarría et al., 2003). Thus, these organizers, secondary to those that operate throughout the embryo during gastrulation, usually develop within the previously broadly regionalized neuroectoderm at given genetic boundaries (frequently where cells expressing different transcription factors are juxtaposed). Their subsequent activity refines local neural identities along the AP or DV axes and regionalizing the anterior neural plate and neural tube (Meinhardt, 1983; Figdor and Stern, 1993; Rubenstein and Puelles, 1994; Shimamura et al., 1995; Wassef and Joyner, 1997; Rubenstein et al., 1998; Joyner et al., 2000).

Three regions in the neural plate and tube have been identified as putative secondary organizers: the anterior neural ridge (ANR) at the anterior end of the neural plate, the zona limitans intrathalamica (ZLI) in the diencephalon, and the isthmic organizer (IsO) at the mid-hindbrain boundary (Vieira et al., 2010). Therefore, the isthmic constriction contains the IsO (Figures 1D,D′), which has been extensively studied during the last decade (Martinez and Alvarado-Mallart, 1989; for review see Martínez, 2001; Wurst and Bally-Cuif, 2001; Echevarría et al., 2003; Aroca and Puelles, 2005; Hidalgo-Sánchez et al., 2005; Nakamura et al., 2005; Partanen, 2007). It is involved in maintaining the mid-hindbrain boundary and providing structural polarity to the adjoining regions in order to orchestrate the complex cellular diversity of the mesencephalon (rostrally) and the cerebellum (caudally; Itasaki and Nakamura, 1992; Martínez, 2001; Rhinn and Brand, 2001; Crespo-Enriquez et al., 2012).

The earliest molecular event for the IsO specification is the differential expression in the neural plate of *Otx2* in the rostral epithelium and a *Gbx2* in the posterior domain (Wassarman et al., 1997; Shamim and Mason, 1998; Broccoli et al., 1999; Katahira et al., 2000; Figures 1B,C′, 4). In the avian embryo at HH8 an *Otx2* and *Gbx2* negative neuroepithelial gap separate these domains, but at stages HH9–10 they come to overlap across the prospective mid-hindbrain boundary (Garda et al., 2001). Some of the key experiments, revealing the molecular nature and regulation of the signals for the specification of the IsO, were performed already 18 years ago. A member of the fibroblast growth factor (FGF) family, *Fgf8*, was found to be highly expressed in the most anterior hindbrain (Heikinheimo et al., 1994; Crossley and Martin, 1995; Figure 1D). Furthermore, beads-containing FGF8 protein were found to effectively mimic the activity of the IsO tissue when transplanted either into the diencephalon or posterior hindbrain (Crossley et al., 1996; Martinez et al., 1999). Since these experiments, members of the FGF8 subfamily (Itoh and Ornitz, 2004) have been shown to be also morphogenetic signals that regulate structural aspects of midbrain, isthmus (Isth), and r1 development (Figures 1A,D,D′).

*Fgf8* expression is first activated at HH9+ in birds and at E8.5 in mice at the interface of *Otx2* and *Gbx2* positive neuroepithelial cells. WNT1 and EN2 proteins are already expressed at this stage across the incipient boundary, with a maximum expression level at the *Fgf8* positive domain, showing decreasing gradients oriented either rostrally toward mesencephalic epithelium or caudally toward rhombencephalic epithelium, respectively. The co-expression of *Otx2* and *Gbx2* in the IsO territory essentially disappears by HH11–12 and both domains become thereafter mutually excluded and complementary (Millet et al., 1999; Garda et al., 2001; Liu and Joyner, 2001). The caudal limit of *Otx2* expression and the rostral limit of *Gbx2* therefore mark the mid-hindbrain molecular boundary (MHB; Millet et al., 1996; Hidalgo-Sánchez et al., 1999; Martínez, 2001). Secondarily, *Lmx1b* and *Wnt1* are co-expressed in a thin band confined to the caudal most *Otx2* expression domain, abutting the *Fgf8* domain at the rostral most edge of the hindbrain. *Lmx1b* activates *Wnt1* in a cell-autonomous manner and represses *Fgf8* in a non-autonomous way, thus contributing to maintain the rostral limit of *Fgf8* at the MHB (Matsunaga et al., 2002) and thus being essential for the initial steps of mid-hindbrain development (Guo et al., 2007). Note that although early *Fgf8* expression appears in the territory co-expressing *Otx2/Gbx2*, double deletion of these two transcription factors in the mouse does not affect the activation of *Fgf8* expression (Li and Joyner, 2001; Martinez-Barbera et al., 2001). Other genes expressed at very early stages across the prospective MHB, such as *Pax2* (Rowitch and McMahon, 1995; Joyner, 1996; Hidalgo-Sánchez et al., 2005) and *Iroquas* (*Irxs*) seem required for the expression of *Otx2*, *Gbx2*, and *Fgf8* and the proper formation of the mesencephalic and rhombencephalic vesicles (Vieira et al., 2010). Recently it was proposed that *Gbx2* and *Fgf8* are sequentially required for formation of the mid-hindbrain boundary, playing a crucial role in maintaining here a boundary of cell lineage by restricting cell movement (Sunmonu et al., 2011; Figure 4).

Moreover, FGF8 signal may act at the IsO in concert with other signaling molecules, such as WNT1, Sonic Hedgehog (SHH) and transforming growth factor (TGF)-β family members (Danielian and McMahon, 1996; Matsunaga et al., 2002; Vogel-Höpker and Rohrer, 2002; Castelo-Branco et al., 2003; Farkas et al., 2003; Blaess et al., 2006). The morphogenetic activity of the IsO is then a consequence of a specific temporo-spatial expression of molecular signals, which regulate the specification and structural development of mesencephalic and cerebellar neuroepithelial territories. Alterations of *Fgf8* and *Gbx2* gene expression lead to massive disruption of the mid-hindbrain neural territory by gene patterning dysregulation (Wassarman et al., 1997). A decreasing gradient of FGF8 protein concentration in the alar plate of the isthmus and r1 is fundamental for cell survival and the differential development of cerebellar regions (Chi et al., 2003; Nakamura et al., 2005; Basson et al., 2008). In the basal plate, FGF8 gradient is crucial for cell survival and, together with SHH, essential for the development of caudal serotonergic and rostral dopaminergic fates of progenitor cells, as well as the localization and development of other basal derivatives, such as noradrenergic cells in the locus coeruleus (in the rhombencephalon) and the red nucleus (in the mesencephalic tegmentum; Wurst and Bally-Cuif, 2001; Chi et al., 2003; Puelles and Rubenstein, 2003; Prakash and Wurst, 2006; Prakash et al., 2006). On the other hand, mesencephalic and diencephalic epithelia are also receptive to FGF8 (Martinez et al., 1991, 1999; Crossley et al., 1996; Crespo-Enriquez et al., 2012), which possibly regulates gene expression and neuroepithelial polarity in the alar plate of these territories (Vieira et al., 2006; Crespo-Enriquez et al., 2012).

Finally, the proposed mechanism by which FGF8 signaling spreads over a field of target cells, at least in zebrafish, is established and maintained by two essential factors: firstly, free diffusion of single FGF8 molecules away from the secretion source through the extracellular space and secondly, an absorptive function of the receiving cells regulated by receptor-mediated endocytosis (Yu et al., 2009; Nowak et al., 2011; Müller et al., 2013). Several studies have disclosed the position preferences of neuroepithelial cells to FGF8 planar signal activity. The differential orientation and polarity of the FGF8 signal seems to be directly dependent on the spatial position of mouse Fgf8-related secondary organizers and on the activity of the negative modulators, *Mkp3* (Figures 1F,F′; Echevarria et al., 2005a,b), *Sef* (Fürthauer et al., 2002; Tsang et al., 2002), and *Sprouty1/2* (Spry1/2; Minowada et al., 1999; Echevarria et al., 2005b; Figure 4). Relevant published findings in chick embryos claimed that FGF8b may also translocate into the nucleus, and this nuclear FGF8b could function as a transcriptional regulator to induce *Spry2* in the isthmus independently of ERK phosphorylation (Suzuki et al., 2012). Similar findings in mouse found maintenance of *Spry2* expression pattern along the Isthmic region in temporally absence of FGF8 in the extracellular compartment, as well as ERK phosphorylation (Crespo-Enriquez et al., 2012). The latter findings reaffirm the existence of positional information encoded by the FGF8 signal through planar transcellular corridors in neuroepithelial cells along the vertebrate neural tube.

## Histogenesis and cellular identity of the cerebellar anlage

**Figure 2:**
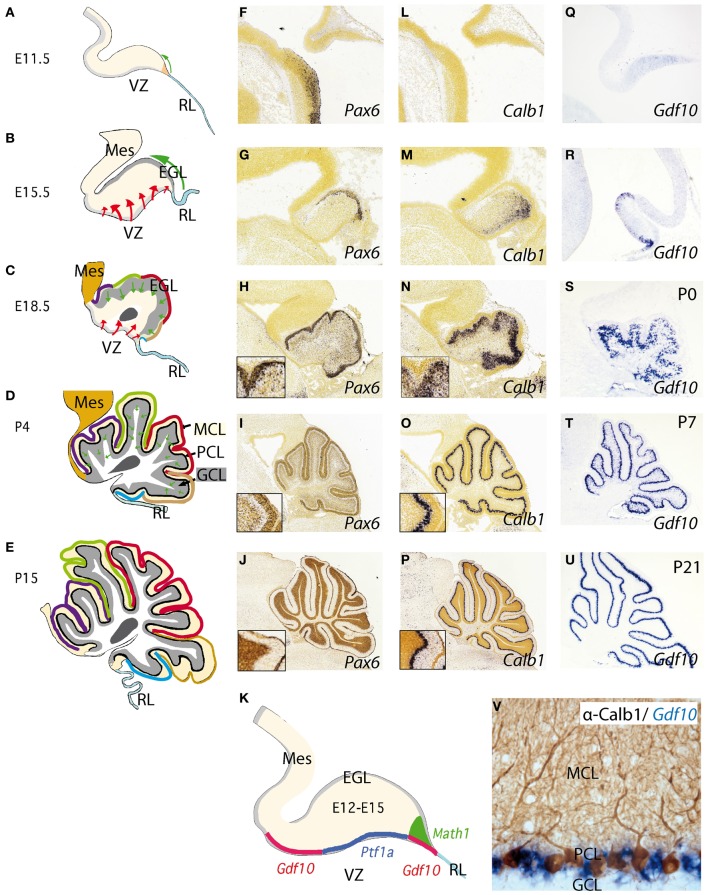
**Development of the cerebellum from the rhombencephalic alar plate from early stages to adulthood. (A–E)** Cerebellar morphology/anatomy at different stages of development. The cerebellar folia development is showed from E11.5 onwards identifying the corresponding folia primordia bulges as color code lines from rostral to caudal (see also Sudarov and Joyner, 2007). Tangential migration from the rhombic lip (RL) is indicated as a green arrow corresponding to excitatory granule precursor cells that are specified by the expression of *Math1* **(K)**. These cells build up the external granule cell layer and from neonatal stages to P14, they descend radially forming the internal granule cell layer (GCL) located below Purkinje cell layer (PCL). The red arrows highlight the radial migration of cells originating from the ventricular zone (VZ). This is where Purkinje cells (PCs) and GABAergic cells of the cerebellum are born and specified by the early expression of *Ptf1a* **(K)**; see also Dastjerdi et al. (2012). (**F–U**) Represent *in situ* hybridizations (ISH) of corresponding markers for each cerebellar cell type. *Pax6* labeling granule cells (**F–J**). From **(H)** to **(J)** the insert shows the position of *Pax6*-positive cells at the EGL and later on, in the GCL **(J)**. In the same manner (**L–P**) represent *Calbindin* (*Calb1*) showing the early location of PCs and their migration from the VZ to the final Purkinje cell monolayer, PCL (see also Sotelo, 2004). (**Q–U**) shows the expression pattern of *Gdf10* (a marker for Bergmann glial cells) by means of ISH during cerebellar development. **(V)** Here we show the expression of *Gdf10* in Bergmann glial cells in adult mice, together with Calbindin- positive Purkinje cells. **(K)** represents the molecular specification of the different cell types (excitatory vs. inhibitory) in the cerebellum including the two germinal centers ventricular zone (VZ) and rhombic lip (RL). Panels (**F–J**) and (**L,M**) were taken from Allen Institute for Brain Science public resources (http://www.brain-map.org/).

**Figure 3:**
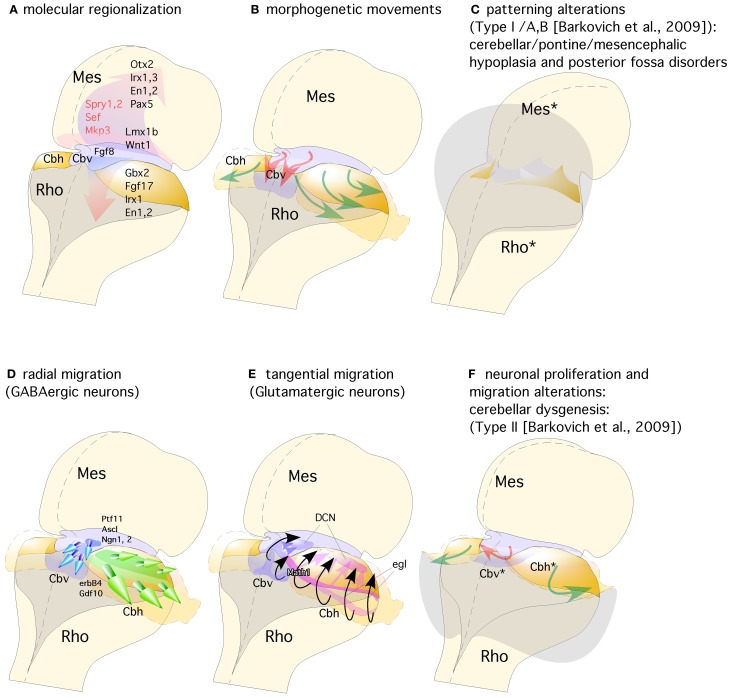
**Representation of a dorsal view of mid-hindbrain junction, where the isthmic segment is colored in light purple (which will generate also cerebellar vermis: Cbv) and the cerebellar plates in yellow (which will generate cerebellar hemispheres). (A)** The isthmic organizer expressing *Fgf*8 (pink arrows) induces the expression of *Sprouty*, *Sef*, and *Mkp3* in this region; and is also required for other genes differentially expressed in the midbrain (Mes) or rhombencephalic (Rho) neuroepithelium. **(B)** The spatio-temporal expression of these genes regulates the normal morphogenesis and growth of the cerebellar vermis (Cbv; red arrows) and hemispheres (Cbh; green arrows). **(C)** Failure in proper isthmic organizer development (due to a lack of morphogenetic signaling or disruption of gene expression) can result in cerebellar (Rho^*^) and mesencephalic (Mes^*^) hypoplasia due to a strong increase of cell death with posterior fossa disorders and fourth ventricle dilatation (gray shadow). **(D)** Radial migration of GABAergic neurons in the cerebellar vermis (Cbv; blue arrows) and hemispheres (Cbh; Green arrows). **(E)** Rhombic lip specification is regulated by *Math1*. Tangential migration of glutamatergic neurons of the deep cerebellar nuclei (DCN) and granule cells (egl) are represented by pink and black arrows, respectively. **(F)** When normal development of cortical cerebellar cells is disrupted, the structural phenotype is classified as cerebellar dysgenesis (Cbv^*^ and Cbh^*^), with enlargement of the fourth ventricle and reversion of cerebellar-choroidal junction (arrows).

The cerebellum is indeed a unique brain structure dependent of FGF8 signal and *Gbx2* homeobox expression. The medial part is know as the vermis and develops from the isthmic and r1 roof plates, while the lateral parts are know as the cerebellar hemispheres and develop from the cerebellar plates at the r1 alar region (Figures 3A,B; Martinez and Alvarado-Mallart, 1989; Hallonet et al., 1990; Sotelo, 2004; Zervas et al., 2005). The cerebellum is further divided into cortex, white matter, and cerebellar nuclei. The cortex occupies the entire surface and is greatly increased in extension by the characteristic lobulations and foliations, which from a midsagittal section have the appearance of a “tree” (Figure 2E). The cerebellar foliation is consequence of mechanical forces that induce fissure formation (see color-coded folia identification formation in Figures 2C–E). It has been proposed different causal mechanisms for cerebellar foliation: one possibility for folia formation in the cerebellum is because Purkinje cells (PCs) anchors the cortex to the underlying white matter via their axons at positions that define the base of fissures (Altman and Bayer, 1997). Alternatively, differential rates of granule cell precursor proliferation, with highest rates at the base of the fissures, have been suggested to underlie the postnatal growth of folia (Mareš et al., 1970). Recent studies have identified a reproducible series of cellular changes that the three major cerebellar cell types (PCs, granule cells, and Bergmann glia) undergo during initial formation of fissures demonstrating that the timing of these cellular changes governs folial shape (Sudarov and Joyner, 2007). The latter authors proposed a new model for cerebellar cortex folia specification whereby changes in the behavior of granule cell precursors drive the formation of “anchoring centers” at the base of each fissure consisting of PCs, granule cells, and Bergmann glia cells. Then folia outgrowth continues by a self-sustaining process involving the coordinated action of both granule cells and Bergmann glia. *En1/2* homeobox genes have been found to be crucial for the production of the distinct medial (vermal) and lateral (hemisphere) foliation patterns in mammalian cerebellum. Thus, these genes are proposed as a new class of genes that are fundamental for patterning cerebellum foliation throughout the medio-lateral axis acting late in development (Cheng et al., 2010; Orvis et al., 2012).

Another interesting aspect of the cerebellar cortex is its quite stereotyped cytoarchitecture. The neuronal subtypes are connected to each other in the same manner, building a cerebellar microcircuit (see below). However, and despite the well-known participation in coordinating proprioceptive-motor processing, the cerebellum is found to be involved in other very important higher functions such as cognition, emotion, and language processing (Zervas et al., 2005; Barkovich, 2012).

The adult cerebellar cortex is laminated into three layers. The molecular cell layer (MCL), rich in neuropil consisting mainly of parallel fibers, purkinje dendrites, and glial cell processes as well as neurons allocated at superficial and deep zones, such stellate and basket cells (Figure 2D). The PC layer (PCL) is composed of a monolayer of Calbindin positive PCs (Figures 2D,L–P), candelabrum cells (Lainé and Axelrad, 1994), and Bergmann glia (see below; Figure 2V). The final and deepest layer is the so-called granule cell layer (GCL) and is the widest cerebellar layer, mainly composed of Pax6 positive granule cells (Figures 2D,F–K) as well as Golgi, Lugaro, and unipolar brush cells (Sotelo, 2004; Zervas et al., 2005). PCs are the only output of the cerebellar cortex, while inputs coming to the granular cells are transmitted to PCs via their axons, the parallel fibers. On the other hand, inhibitory interneurons such as stellate and basket cells innervate dendrites and soma of PCs respectively. Deeply, with respect to the cortex, the white matter is located in the center of the cerebellum. It extends to the ventricular surface of the 4th ventricle, accommodating the cerebellar nuclei (from medial to lateral: the fastigial, the interpositus, and the dentate nucleus). Axons coming from diverse cerebral origins enter the cerebellum through any of the cerebellar peduncles (superior, middle, and inferior). They project either directly or collaterally to the cerebellar nuclei and to the GCL in the cortex, and are named mossy fibers. Only those axons coming from the inferior olive are called climbing fibers because of their “climbing” features to synapses the PC dendrite arborization (Sotelo, 2004).

Neurochemically the cerebellar cortex contains two glutamatergic neuronal subtypes (granule and unipolar brush cells) and five GABAergic subtypes (Purkinje, Golgi, Lugaro, Stellate, and Basket cells). The deep cerebellar nuclei (DCN; Figure 2C), contain both GABAergic interneurons and glutamatergic projection neurons (Wang and Zoghbi, 2001; Hoshino, 2006; Leto et al., 2006; Carletti and Rossi, 2008). Fate-mapping studies of the developing cerebellum have uncovered when and where cells are born and which migratory routes they follow in order to reach their final position. Cerebellar neurons are generated from two major germinal centers: the external granule layer (EGL) and ventricular zone (VZ; Sotelo, 2004; Millen and Gleeson, 2008; Figures 2B,C). Over the past decades has been proven that granule cells are produced by early granule cell precursors located in the EGL that originate from the rhombic lip (RL; Figures 1A,B,B′, 2A–E, 3D,E), at the interface of the dorsal neural tube and the extended roof plate of the 4th ventricle (the choroid plexus; Chp; Wingate, 2001). Also, the glutamatergic DCN neurons and unipolar brush cells are derived from the RL (Fink et al., 2006; Carletti and Rossi, 2008). Therefore, all glutamatergic neurons in the cerebellum appear to originate from the RL. The anterior RL expresses *Math1* (also called *Atoh1*) as early as embryonic stage E9.5 in mice. *Math1* is induced by bone morphogenetic protein (BMP) from the roof plate, which itself is differentiating into the Chp (Basson et al., 2008; Figures 2A–E). *Math1* positive RL progenitor cells give rise to multiple glutamatergic cell derivatives in a time-dependent sequence. Progenitors of the rostral part of the RL migrate through over the cerebellar anlage and give rise to granule progenitors cells and DCN (Figures 3D,E). The caudal part of RL gives rise to multiple brain stem precerebellar nuclei, including the pontine nuclei and superior and inferior olive. Thus, *Math1* positive RL cells (Figures 2K, 3E) generate cerebellar granule cells which mature in the EGL and later migrate inwards into the definitive granular cell layer in a anterior to posterior temporal gradient (green arrows in Figures 2A–D, 3D,E; Sotelo, 2004). Unipolar brush cells are the last *Math1*-positive RL population migrating through the cerebellar white matter to their final GCL locations (Millen et al., 1999; Bermingham et al., 2001; Wang et al., 2005; Millen and Gleeson, 2008). Yet, during the first 2 postnatal weeks in mice, granule cell precursors continue differentiating and migrating radially through MCL and PCL layers to form the final internal GCL leaving their bifurcated axons in the MCL (the parallel fibers; Hatten and Heintz, 2005).

**Figure 4:**
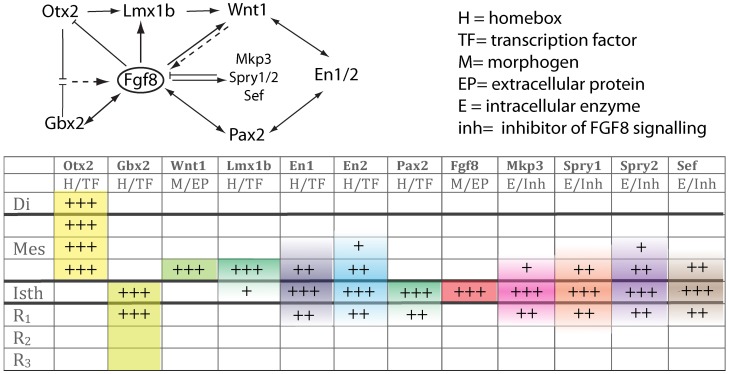
**The upper scheme represents the functional interaction (induction/inhibition) of genes that, together with *Fgf8*, are involved in the molecular maintenance of isthmic region at E9.5**. The table below summarizes the expression intensity and range of genes along the AP axis of the neural tube focusing on the isthmus. The color code depicts their mRNA expression range from the isthmus toward rostral or caudal regions.

The second germinal center, the VZ, has been thought to give rise to cerebellar GABAergic neurons (Altman and Bayer, 1997; Sotelo, 2004; Hoshino, 2006; Sudarov and Joyner, 2007; Carletti and Rossi, 2008; red arrows in Figures 2B,C,K). A recent genetic inducible fate-mapping study demonstrated that dorsal r1 first undergoes an orthogonal rotation such that the anterior posterior axis (A-P axis) of r1 at E9.5 becomes the medio-lateral axis (M-L axis) of dorsal r1 at E12.5 (Sgaier et al., 2005; Figure 3B). The M-L axis of ventricular-derived cells is then retained: PCs generated medially are located in the vermis while laterally generated PCs populate the hemispheres (Sgaier et al., 2005, 2007). The pancreas transcription factor 1 (*Ptf1a*), which encodes a bHLH transcription factor, is expressed at the VZ (Figures 2K, 3D; Hoshino et al., 2005; Hoshino, 2006). The characterization of a novel mutant mouse, *Cerebelless*, which lacks the entire cerebellar cortex but still survives into adulthood, has clarified that *Pft1a* is required for generating all cerebellar GABAergic neurons including the PCs. Thus, *Math1* and *Ptf1a* participate in regionalizing the cerebellar neuroepithelium, and define two distinct territories, the VZ (*Ptf1a* positive) and the upper RL (*Math1* positive), which generate GABAergic and glutamatergic neurons, respectively (Hoshino et al., 2005; Pascual et al., 2007; Figures 2K, 3D,E). The earliest markers for GABAergic PC progenitors express *Neph3*, *E-cadherin* (Minowada et al., 2010) as well as *Corl2* (Minaki et al., 2008) in the ventricular and subventricular zone while in post-mitotic PCs neurons other molecular determinants such as Calbindin, is expressed (Figures 2L–P; Sotelo, 2004; Muguruza and Sasai, 2012). Moreover, the expression domains of three pro-neural genes (*Ascl1*, *Neurog1*, and *Neurog2*) overlap with that of *Ptf1a* in the VZ (Zervas et al., 2005; Zordan et al., 2008; Dastjerdi et al., 2012; Figure 3D). A closer analysis of the role of *Ascl1* in cerebellar neurogenesis, established that *Ascl1* positive progenitors progressively delaminate out of the VZ to settle first in the prospective white matter, and then in the cerebellar cortex (Grimaldi et al., 2009). These authors demonstrated by gain of function experiments of *Ascl1* an increase of *Pax2* positive interneurons and *Olig2* positive oligodendrocyte precursors, while glutamatergic neurons, astrocytes, and Bergmann glial (BG) cells were not affected. On the other hand, the lack of *Ascl1* led to a dramatic reduction of *Pax2* and *Olig2* precursors. Interestingly, no change was found in PC development in any of the experiments mentioned above. Thus, the latter evidence suggests that *Ascl1* contributes to the generation of GABAergic interneurons and DCN as well as to PC development but not to their specification.

In addition to GABAergic neurons, progenitor cells located in the VZ of the fourth ventricle also give rise to BG cells. During development, the processes of BG provide structural support to the expanding cerebellar plate (see below). In addition radial Bergmann fibers act as essential guide rails for the migration of granule cells (Rakic, 1990) and contribute to the elaboration of PC dendrites (Yamada et al., 2000) and stabilize synaptic connections onto these neurons (Iino et al., 2001). Indirect evidence suggests that neuregulin, a member of the epidermal growth factor family, and its membrane receptor erbB4 are involved in the cerebellar induction of the radial glial scaffolds for granule cell migration (Rio et al., 1997). Migrating granule cells, as well as their EGL precursors (green arrows in Figures 2A–D), express neuregulin, whereas Bergmann fibers express erbB4 in the postnatal cerebellum. Moreover, activation of the receptor with soluble neuregulin mimics the effects of neuron–radial glial interactions in the induction of radial glial formation. In contrast, when the glial erbB4 receptors are inactivated by transfection with a dominant-negative form of erbB4, granule cells and soluble neuregulin fail to induce the radial glial phenotype *in vitro* (Rio et al., 1997).

BG cells, like interneurons and PCs, are born in the VZ of the fourth ventricle, where they express among other lineage-restricted markers growth and differentiation factor 10 (*Gdf10*; Figures 2Q–V; Alcaraz et al., 2006; Koirala and Corfas, 2010). In mice *Gdf10* was identified as a marker expressed in the PCL of the cerebellum (Zhao et al., 1999). Based on a publication by Zhao and colleagues, *Gdf10* is expressed in PCL but not in GCL. Finally, it was further demonstrated that *Gdf10* is expressed in BG cells (Koirala and Corfas, 2010). *Gdf10* localizes to the cerebellar VZ as early as E13.5 and by E15.5, *Gdf10*-positive cells actively migrate toward the pial surface, as part of a migration process that these cells undergo until postnatal day P7 (Yamada and Watanabe, 2002). From P7 onwards *Gdf10* expression in the PCL becomes progressively more restricted to fine band of BG cells located between the soma of Purkinje neurons (Figures 2U,V). Our knowledge of possible interaction partners of *Gdf10* is very limited and *Gdf10* null mutant mice develop normally (Zhao et al., 1999). However, in PC degeneration mice (pcd3J) *Gdf10* is reduced to 15% of the signal obtained from wild type littermates of the same age (4 postnatal months; Rong et al., 2004).

## Human cerebellar disorders related to defects at the isthmic organizer

As we mentioned above, the cerebellum and its stereotyped circuitry, contributes not only to motor learning and correction of motor acts, but also to cognitive and emotional functions. Clumsiness and abnormal motor behavior have been well-documented in disorders such as autism and Asperger's syndrome (see Frith, 1991; Fatemi et al., 2012; Rogers et al., 2013), in dyslexia (Nicolson et al., 2001) and in schizophrenia (Owens et al., 1982). The cerebellum is ontogenetically and functionally heterogeneous, with cerebellar zones from different precursor domains (r0/r1 vermis or r1 cerebellar hemispheres) selectively interconnecting with different cerebral subsystems. In addition, the main cellular and molecular processes in cerebellar histogenesis are regulated by the same morphogenetic signals operating in other brain regions (Airey et al., 2001; Echevarría et al., 2003; Vieira et al., 2010; Figures 3A,B). Thus, it is not surprising to find developmental disorders that affect both different functional systems in the forebrain and the cerebellum. Advances in developmental genetics, neurobiology, molecular biology, and neuroimaging have led to a better understanding of developmental disorders arose from the embryonic midbrain and hindbrain, (Barkovich et al., 2007, 2009). Although malformations of the hindbrain maybe the only recognized abnormality in individuals with mental retardation or autism (Soto-Ares et al., 2003; Courchesne et al., 2005), they are more commonly associated with malformations of the cerebrum. A contribution to our incomplete knowledge of the clinical consequences of hindbrain and cerebellar anomalies may be due to intrinsic difficulties in neuroimaging and anatomical complexity of the cerebral region.

The combination of a shortened midbrain and/or elongated pons is associated with an enlarged anterior vermis in humans presumably due to a rostral displacement of the IsO, with loss of mesencephalic tissue and gain of cerebellar tissue (Figure 3C). In fact, this malformation is presumed to result from *GBX2* predominance over *OTX2* and a consequent rostral shift of the IsO (Chizhikov and Millen, 2003; Barkovich et al., 2009). In the counterpart of this phenotype, Ballabio and co-workers (Quaderi et al., 1997) described opposite phenotype in the Opitz G/BBB syndrome (OS), a X-linked genetic anomaly that is caused by a loss of function of the *MID1* gene. Neuroimaging of human brain patients lacking this gene showed hypoplasia of the anterior cerebellar vermis (Pinson et al., 2004; Fontanella et al., 2008; Figures 3C,F). Concomitantly, *Mid1*-null mice show also vermis hypoplasia among other motor coordination defects (Lancioni et al., 2010). In these mice a rostralization of the mid-hindbrain constriction occurs together with a down-regulation of Fgf17, an important signal during the morphogenetic activity of the IsO (Xu et al., 2000; Zanni et al., 2011). Thus, the increasing of knowledge in basic embryology, genetics, and in cellular and molecular biology of the developing brain must be emphasized to prove the importance in recognizing, understanding, and classifying anomalies in human pathologies (Barkovich, 2012).

### Conflict of interest statement

The authors declare that the research was conducted in the absence of any commercial or financial relationships that could be construed as a potential conflict of interest.
